# Positive Sentiment and the Donation Amount: Social Norms in Crowdfunding Donations During the COVID-19 Pandemic

**DOI:** 10.3389/fpsyg.2022.818510

**Published:** 2022-02-21

**Authors:** Yan Peng, Yuxin Li, Lijia Wei

**Affiliations:** Department of Mathematical Economics & Mathematical Finance, Economics and Management School, Wuhan University, Hubei, China

**Keywords:** crowdfunding, social norms, sentiment analysis, COVID-19, donation

## Abstract

Public welfare fundraising has been used to collect donations for medical supplies and has played an important role in the fight against the COVID-19 pandemic. This paper studies online crowdfunding donations from the Alumni Association of Wuhan University to North American alumni; donation data are used to investigate how individuals' donation behavior is affected by the previous donation amount and information provided by the fundraising platform. First, our results show that one's donation amount is positively affected by the previous donation amount. Second, the donor's positive sentiment in the message that he or she leaves, as measured by either natural language processing or a manual rating, can affect the subsequent anonymity and messages but not the subsequent donation amount. Third, anonymous donations are much smaller than non-anonymous donations.

## Introduction

Online public welfare fundraising, in which donors are purely donating and asking for nothing in return, is gradually replacing on-site fundraising because of its advantages of faster dissemination, its ability to reach a larger audience, and its greater pertinence. In 2020, 12.9% of total fundraising came from online giving worldwide, and the growing trend of online donations was clear^1^. Online crowdfunding is an important fundraising tool launched on an online platform within a certain period of time to obtain small donations from a group of people who mostly do not know each other (Mollick, 2014).

In economics, people are often assumed to be self-interested, but in reality, human is not entirely driven by material interests. Exploring donors' motivations and identifying the determinants that affect donation behaviors are essential and practical for fundraisers to increase the donation amount. Bagheri et al. (2019) explore the motivations of donors to fund projects on charity crowdfunding platforms and suggest a set of intrinsic individual motivations, including shared problems, values, thoughts, and beliefs, helping a minority, technical knowledge, and the capacity of the project to learn from and help to realize ideas and create value, that lead to donations on charity crowdfunding platforms. Lee and Chang (2008) point out the intrinsic determinants of charity behaviors, including psychographic, and attitudinal factors, such as general perceptions of charities, a sense of social responsibility, familiarity with a charity, and empathy. ^1^

Many studies have been conducted to explore what kinds of information and the extent to which the information can mediate individuals' intrinsic determinants and further nudge individuals' donation behaviors. With the development of the internet and the changes in charity crowdfunding channels, online charity platforms offer opportunities for fundraisers to provide potential donors with information that could influence their behaviors. It is of great theoretical and practical significance for researchers, fundraisers, and charity platforms to address whether several common types of information and the basic design of many online charity platforms positively impact individuals' donation behaviors.

This paper uses data from online crowdfunding donations by Wuhan University alumni during the COVID-19 pandemic to study the factors affecting crowdfunding donations. The Wuhan University Alumni Association launched the “Donate Masks for North American Alumni” donation campaign in March 2020 to purchase epidemic prevention materials to assist alumni overseas.^2^ The crowdfunding campaign used the WeChat public platform to openly collect donations and opinions from a large number of alumni.

The length of the single donation sequence in this study is close to 1,500, and half of the donors left a message, which allows us to use linguistic sentiment analysis^3^ for the sequence. To analyze the impact factors in the process of online crowdfunding donations, this paper includes historical donation amounts and the lengths and sentiments of messages in econometric models. In addition, we study how historical donation information affects the anonymous selection of subsequent donors, which is another topic not considered in the previous empirical literature. Our research demonstrates the role of the message, atmosphere, donation amounts (descriptive social norm), and anonymity behaviors of previous donors in subsequent donors' behaviors.

In a disaster that affects a wide range of areas and a large number of people, people can share experience messages online and quickly allocate social resources through online crowdfunding donations, which is especially important and effective. The features of this kind of donation can provide insights for research focusing on individuals' donation behaviors in charitable crowdfunding projects launched to fight against disasters such as the COVID-19 pandemic. The donation data also allow us to focus on how information affects individuals' donation behavior, controlling all donors with the same social identity and similar educational background.

This paper confirms the impact of descriptive social norms on crowdfunding donations. As some of the earliest researchers on leaving messages in donations, we do not find evidence that leaving messages and donors' sentiments can affect the subsequent donations amounts. These results are very similar regardless of whether we use natural language processing or a manual rating. However, Saleh et al. (2021) found that crowdfunding donations related to the COVID-19 pandemic have significantly longer descriptive messages, more social media sharing, and a higher total donation and last longer than other donations. Our paper points out the possibility that emotional messages that are left may promote enthusiasm for participation (the total number of participants) in donations but does not increase the average donation amount.

This paper is organized as follows. We first outline the literature review and hypothesis development, second describe the data and method, third present the results, and finally offer the discussion and conclusion.

## Literature Review and Hypothesis Development

### Influence of Social Information on Donation Behavior

Online donation platform practitioners often apply information intervention to encourage visitors to donate more. Much research focuses on what kind of information can nudge individuals' donation behaviors, among which the information and donation behaviors are usually related to individuals' donation amount.

However, the conclusions regarding the positive or negative impacts of information on donation behaviors are not consistent. Many prior studies show that information about the previous donation amounts increases individuals' donation amounts (Shang et al., 2007; Martin and Randal, 2008; Shang and Croson, 2009; Smith et al., 2015; Goeschl et al., 2018; Vesely and Klöckner, 2018; van Teunenbroek and Bekkers, 2020; Drouvelis and Marx, 2021; Li et al., 2021; van Teunenbroek et al., 2021). There are also several studies drawing different conclusions and showing a negative effect (Croson and Shang, 2008, 2013; Meyer and Yang, 2016; Kubo et al., 2018); or no effect (Murphy et al., 2015) of several types of social information on donation amounts under certain situations.

Some studies explore the mechanism of the impact of social information on donation behaviors. Smith et al. (2015), Sasaki (2018), and van Teunenbroek and Bekkers (2020) suggest that social information influences donation or contribution behavior *via* social norms, which are a standard or reference for what is appropriate, and then triggers subsequent decision makers' conformity behaviors. Different from their conclusion, van Teunenbroek et al. (2021) find no evidence that social information affects giving behavior or mood *via* perceived social norms.

Descriptive social norms are an essential category of social norms and have attracted much attention in the literature. Using field experiments, Agerström et al. (2016) and Bartke et al. (2017) find that providing people with descriptive norms (e.g., “this is what most people do,” or “2/3 of the population in Germany make charitable donations each year”) substantially increased charitable giving. Goette and Tripodi (2020) find that donors in the experiment expect others to donate more, and in turn, they donate more themselves. This phenomenon is described as the social information effect (Shang and Croson, 2009; van Teunenbroek and Bekkers, 2020). The empirical literature finds that online donations can produce descriptive social norms of the donation amount (Smith et al., 2015; Sasaki, 2018). In the crowdfunding donations examined in this paper, the donation page displays only the last five donation amounts, which is convenient for investigating the impact of descriptive social norms.

Since online crowdfunding is a sequential donation process, historical donation information can affect the behavior of future donors (Potters et al., 2005; Gaechter et al., 2010). Meer (2017) also finds that matching grants to donation amounts from a third party, as well as amount competitions among donation projects, could increase the contributions. Based on previous studies, van Teunenbroek et al. (2020) report that descriptive social norms will motivate people's donation behavior through awareness of the need for help as well as perceived descriptive social norms of the donation amount, but at the same time, the donation will become less attractive when the impact of the individual donation, which is also reflected in descriptive social norms, is considered low. Online crowdfunding typically allows people to leave messages; however, no linguistic analysis of donors' messages has been conducted in the previous literature.

On the internet, one can more easily display all kinds of information to affect other people's decisions. In an online crowdfunding donation, donors can choose whether to provide personal information and/or leave a message, and future donors can see the information left by the previous donors and their donation amount. A fixed environment for donating and expressing opinions on the website can help us observe the channels that influence donations.

We have reasons to believe that messages and the atmosphere in the donation may also impact behavior. This inference is based on the following three facts. First, studies show that people must be aware of there being a need for help before they feel motivated to give (Levitt and Kornhaber, 1977; Bekkers and Wiepking, 2011); the longer the message, especially the sentiment shown in the message, the greater the potential donors' perception of need. Second, positive sentiment has a stimulating effect. Many positive messages reflect that a donation is attractive and can be regarded as a signal of the high quality of the donation project, which may motivate people to donate (Quinn and Dutton, 2005). Third, positive sentiment has a strengthening effect. Psychological research has shown that people tend to repeat actions that make them feel positive (Collins, 1993). Therefore, historical messages conveying positive sentiment are likely to inspire subsequent donors to leave messages with positive sentiment. However, there is no literature linking positive sentiment to people's donation behavior because the existing research has not introduced an index for language sentiment tendentiousness.

Our work contributes to the literature on the impacts of social information on donation behavior. The first reason it does so is that previous studies do not draw a consistent conclusion on whether social information has a positive or negative impact on donation behavior, especially the donation amount. In addition, when exploring what kind of information can nudge individuals' donation behaviors, prior studies consider only the information and behavior related to the donation amount, and they pay less attention to the donation message, even though the message holds great significance for fundraisers, donors, and recipients when funds are raised for people facing a disaster.

### Anonymity, Social Norms, and Donation Behavior

Decision observability or unobservability is an essential contextual factor in donation projects. Many field experiments have noted that when donations are non-anonymous, people donate more money than when donating anonymously (Soetevent, 2005; Alpizar et al., 2008; Vesely and Klöckner, 2018). In these field experiments, people were randomly assigned to a charitable donation in either the non-anonymity (also called behavior observability) or anonymity condition.

In this paper, however, donors themselves could choose to be non-anonymous or anonymous on an online sequential donation platform, which means that they had the option to hide their name and avatar or not. Past studies based on these similar anonymous behaviors point out that the most common reason driving people to donate anonymously was to avoid judgments from the public (Peacey and Sanders, 2013; Raihani, 2014; Imada, 2020; Raihani and Power, 2021).

Firmansyah and Pratama (2021) compare donors' anonymity and donation amount on GoFundMe and Kitabisa, donation-based crowdfunding platforms in the United States and Indonesia, respectively, and they find that anonymous and self-identified donors donate a similar amount of money on GoFundMe, while anonymous donors donate significantly less money than self-identified donors on Kitabisa. They attribute the differences in donation and anonymity behaviors between the two donation-based crowdfunding platforms to cultural and religious influences. Individuals in the United States, which is an individualistic country, are more likely to embrace differences. In contrast, individuals in Indonesia are expected to conform to social norms because they come from a collectivistic country.

China is a country dominated by collectivism, and people have been educated to be united since childhood. Under such a social background, we expect our anonymity and donation amount results to be similar to those of Indonesia in Firmansyah and Pratama (2021). We propose the hypothesis that a considerable number of people will choose to be anonymous and that individuals who choose to be anonymous will donate less than non-anonymous individuals.

### Online Crowdfunding Donations (Especially for the COVID-19 Pandemic)

Donation-based online crowdfunding has become an increasingly popular tool because of its time and cost efficiency in obtaining financial support for people facing unexpected events such as natural disasters and pandemics (Sura et al., 2017; Radu and McManus, 2019; Saleh et al., 2021). The emotion and sentiment involved play an essential role in appealing to potential donors to contribute (Korolov et al., 2016; Rhue and Robert, 2018), especially on online charity platforms.

Many social context-related factors impact donors' psychological states and behaviors (Ferguson et al., 2015; Braun, 2017). Li et al. (2021) suggest that participants' social anxiety decreased along with the abatement of the pandemic and that social anxiety completely mediated the relationship between pandemic abatement and the decrease in the contagion of positive donation behaviors. By comparing COVID-19-related campaigns and non-COVID-19-related campaigns, Saleh et al. (2021) suggest that COVID-19-related campaigns raised more money, had a longer narrative description, and were more likely to be shared on Facebook than other campaigns in the study period.

The donation in this paper has characteristics that are similar to those in the literature: a long duration, many participants, messages that are left, and a high total donation. We observed a considerable amount of sharing in the Wuhan University Alumni WeChat group, but we do not have social media sharing data.

### Hypothesis Development

Based on the literature, we construct the following three hypotheses:

**Hypothesis 1 (Message and Sentiment Effect)** *In sequential (crowdfunding) online donation, the length and the positive sentiments expressed in the previous messages can affect those of subsequent donors*.

Previous studies show that the text or video of the project descriptions or charity advertising applied by fundraisers can evoke individual emotions and influence the decision-making of potential donors (Chen et al., 2021; Wymer and Gross, 2021). Based on this idea, we believe that donor messages can also affect donor behavior by arousing the emotions of subsequent donors. Different from previous studies, our paper focuses on the information and emotional transmission between donors instead of focusing on the information and emotion communication among fundraisers and potential donors, as done by previous studies.

In the empirical section, we try to determine whether previous messages and their sentiments affect subsequent messages and sentiments.

**Hypothesis 2 (Descriptive Social Norms)** *In sequential (crowdfunding) online donations, the previous donation amounts can affect the donation amounts of subsequent donors*.

Social norms also include how much others donate. Individuals tend to imitate and follow the observed donation amounts of other donors. As a result, donors may adjust their donations according to the amounts given by previous donors.

**Hypothesis 3 (The Anonymity Effect)** *In sequential (crowdfunding) online donation, previous anonymity can increase the possibility of anonymous subsequent donors. Moreover, anonymous donations are smaller than non-anonymous donations*.

The donor's intention to remain anonymous is also affected by how many previous donors chose to remain anonymous. Economists note that donors are influenced by the estimated impact of their donation (Duncan, 2004). When people choose to be anonymous, their individual impact, or the social norms' impact, is weaker than if they were not anonymous; thus, their willingness to donate will be lower (Firmansyah and Pratama, 2021).

## Data and Method

### Introduction of the Donation Platform and Sample Selection

The fundraising page displays the total amount of funds raised, the total number of donors, and detailed donation information about the last five donors, including their names,^4^ WeChat avatars, donation amounts, and messages left. Donors can choose to remain anonymous, and if they do so, their WeChat avatar will be replaced by a picture showing a pink heart. Additionally, their nicknames will be uniformly displayed as “caring people,” while the display of their donation amount and message will not be affected by their anonymity decision.

**Figure 1:**
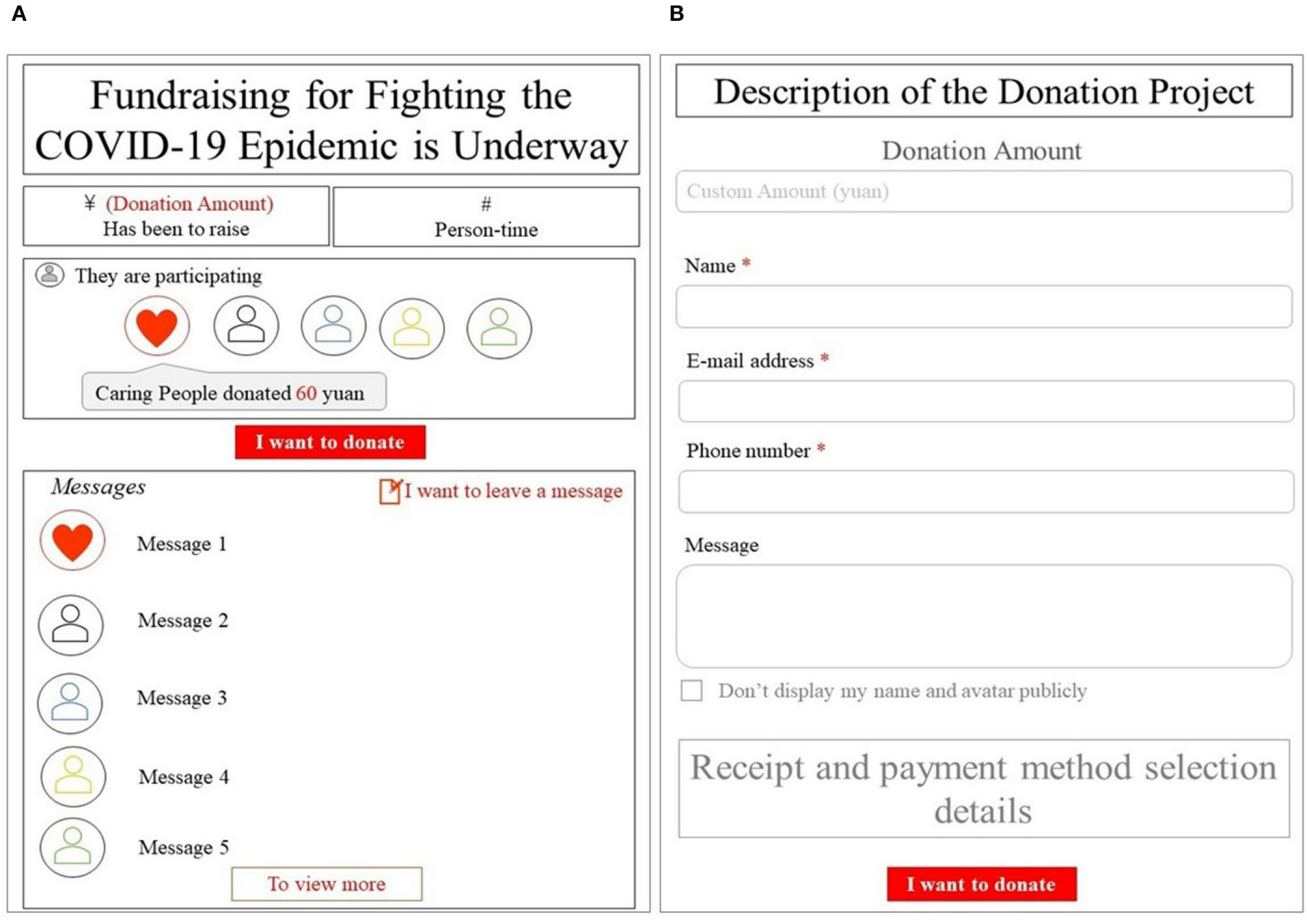
The crowdfunding donation, **(A)** Page 1, **(B)** Page 2. This figure contains the core content of the actual fundraising pages.

As shown in Figure 1A, a person who enters this page can click the red button in the middle of the page, “I want to donate,” to make a donation. Once a donor clicks “I want to donate,” the donation website switches to the second page shown in Figure 1B. After entering the donation amount, filling in his or her private information (including his or her name, email, and phone number), leaving a message (or not), and deciding whether to be anonymous, the name, donation amount, anonymity and message will be updated on the donation page in real time, as shown in Figure 1A.^5^

The donation platform requires real-name authentication; thus, the private information of donors must be submitted. The message is optional for donors, and the donation platform does not set a default message. If a donor does not leave a message, nothing will be displayed in the corresponding place in Figure 1A.

**Figure 2:**
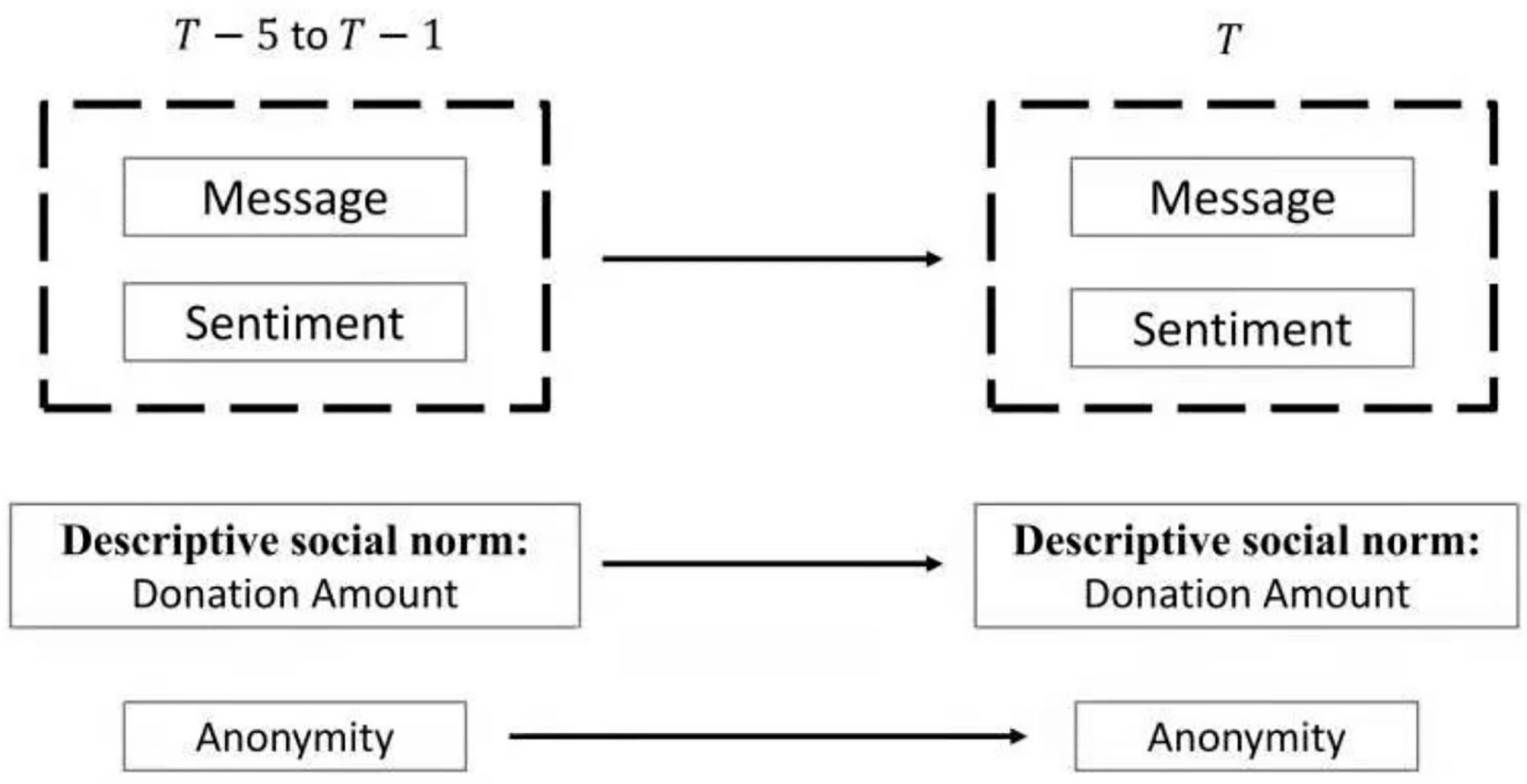
Effect of norms on donations and positive sentiment.

This online fundraising process is a sequential donation; the information of donors has a cascade effect. That is, historical donation information plays a role in the current donation decision, and current donation information affects the behavior of future donors. Specifically, as shown in Figure 2, the information from the previous set of donors includes the previous donation amounts, messages, and positive sentiment reflected in their messages. Assume a donor at time *T* can see the information set and donation amounts in the last five donors *T* −5 to *T* −1. After observing the information of the latest five donors, a donor at time *T* can choose how much to donate, whether to remain anonymous, and what message (if any) to leave.

To avoid the impact of a significant change in the epidemic over a long period of time, we used donation data from the week following the project launch date (from 25 March 2020 to 1 April 2020). This project was launched on the evening of 24 March 2020 and was shared with alumni starting on 25 March 2020. There were several test records created by programmers at the beginning; thus, we exclude the records created on 24 March 2020. Although the donation website was open until early May 2020, the number of donations after April 1 was very sparse and <10. Finally, a total of 1,481 valid samples were obtained, and the total donation amount was 453,249.9 RMB (~65,000 USD).

We collected all available information, including donors' names, donation amounts, messages, and whether the donors chose to remain anonymous. Donors used the messages to express appreciation for the crowdfunding donation, to express optimism about the prospects of fighting the epidemic, or to note deep feelings between the donor and the recipient. There were 718 donors who left messages, accounting for 48% of the total donors. There were some identical messages and a total of 580 different messages.

The donations for these 1,481 samples range from 1 to 15,520 RMB (~2,300 USD), and the average donation amount is ~306 RMB (~42 USD). We use dummy variables to indicate whether a donor chose to remain anonymous: the variable *Anonymous* is equal to 0 for a donor who is not anonymous, and *Anonymity* is equal to 1 for anonymous donors. A total of 475 donors chose to remain anonymous, while 1,006 donors decided to leave their names.

### Message and Sentiment Score

For the non-empty messages, after deleting meaningless characters, we found that each message had an average of 6 characters, indicating that most of the donors' messages were short texts. The overall sentiment of the messages was relatively positive. To determine the positivity^6^ of each message, we first used the snowNLP package in Python. In Table 1, NLP Sentiment denotes the sentiment score determined by the snowNLP package. Chinese differs from English in that there is no interval between words. Therefore, this package first breaks down each message into words and then evaluates them based on the package's specific wordbook and assigns a total sentiment score to the message. This score is a continuous value between 0 and 1: a higher score means the message is more positive. Finally, we set the length of the message and sentiment score to 0 for observations of donations with no message. If we count only donors who left a message, the average sentiment score of the messages is 0.774. Table 1 shows examples of messages and their sentiment scores.

**Table 1:** Message examples in the donation.

**Message example**	**NLP sentiment**	**MR sentiment**
**Example 1:** I am a healed patient of COVID-19. I received many kind people's encouragement and help during the most difficult times. Now it's time to do my part. I hope everyone will unite as one and win this battle against the epidemic. When the spring flowers bloom, we will meet again ~	>0.999	6.556
**Example 2:** The alumni of WHU around the world are one family	0.810	4.167
**Example 3:** Spend together	0.708	2.875

In addition to adopting snowNLP to conduct sentiment analysis, we recruited 51 graduate student subjects (average age = 23.58, 17 males and 34 females) from Wuhan University to rate the donation messages. Every subject was required to rate 116 messages (1/5 of the total) randomly selected from the 580 total unique messages. The subjects were informed of the brief description of the donation projects, and they were informed of the following: “This questionnaire contains 116 questions. Each question stem is a message left by a previous donor when donating. Please rate the emotional strength of each message, with 1 point being the weakest and 7 points being the strongest.”^7^

Finally, we use the average score rated by human subjects as the manual rating (MR) sentiment of each message. Counting only the 580 unique messages, we obtain an average MR sentiment score of 4.289.

The Spearman test results show a significant positive correlation between the NLP and MR sentiment scores (number of observations = 580; Spearman's rho = 0.2409; *p*-value = 0.0000). The MR sentiment scores of the 718 non-empty messages are based on the 580 unique messages rated. Additionally, we set the MR sentiment score to 1 for observations of donations with no message. The descriptive statistics of the MR sentiment scores of 1,481 observations are shown in Table 2.

**Table 2:** Summary statistics.

**Statistic**	**N**	**Mean**	**St. Dev**.	**Min**	**Pctl (25)**	**Pctl (75)**	**Max**
Donation amount	1,481	306.043	569.907	1	100	400	15,520
Anonymity	1,481	0.321	0.467	0	0	1	1
Message length	1,481	6.032	11.434	0	0	9	100
NLP Sentiment	1,481	0.376	0.434	0	0	0.901	1.000
MR sentiment	1,481	2.473	1.676	1	1	4.111	7

We report summary statistics for the main variables in Table 2.

## Results

### Main Results

To study how the behavior of donors is affected by historical donation information, we construct a regression model using ordinary least squares estimation to explore how historical donation amounts, message length, message sentiment^8^ and anonymity affect subsequent donor behavior.

The front page of the crowdfunding platform displays real-time information about the latest five donors. When new donors view the page, they can see the amounts of money donated by the five previous donors before, the content of their messages, and their choice of whether to remain anonymous. If desired, the donors can obtain all the information about the previous donors by scrolling through the pages on their phones. However, due to the limitation of mobile phone interface size, a single page contains information about only five donors at a time, so considerable time and energy are required to obtain more donation information. Therefore, we believe that only the information of the last five donors directly impacts donor behavior; the impact of information from earlier donors is small.

Thus, dynamic regression is conducted according to the following regression equation:


$$\begin{array}{l} Y_{i}=\;\alpha +\beta_{1}\text{log}(\text{DonAmt}5_{i})+\beta_{2}\text{MessLen}5_{i}+\beta_{3}\text{Anonymity}5_{i}\\ \;+\;\beta_{4}\text{Sentiment}5_{i} \end{array}$$

In this regression, the independent variable *log*(*DonAmt*5_*i*_) is the logarithm of the total donation amounts of the latest five donors before the *i*th donor. The reason we use the logarithm value is that donation amounts have a very wide range of values (minimum value, 1 Yuan; maximum value, 15,520 Yuan). *MessLen*5_*i*_ is the total message length of these five donors. We sum the dummy variable values of whether the latest five donors are anonymous to obtain the variable *Anonymity*5_*i*_. In the same way, the total sentiment scores of the five people who left messages before the *i*th donor are calculated as *Sentiment*5_*i*_. The VIF (variance inflation factor) values of *log*(*DonAmt*5), *MessLen*5, *Anonymity*5, and *Sentiment*5 are 1.037, 1.701, 1.016, and 1.675, respectively, which represent a low level of multicollinearity.

Our study aims to determine how an individual's donating behavior is influenced by other people's donation information, specifically how the information of the last five donors affects the subsequent donor's decision to donate. *Y* is the dependent variable of interest, and we consider four dependent variables: the *i*th donor's message length, anonymity, message sentiment score, and donation amount.

The regression results are shown in Table 3. Column (1) shows the relationship between the length of the *i*th donor's message and the donation information of the five donors before him or her. The sentiment scores of the latest five donors have a significant positive impact on the length of the donor's message (*p*-value = 0.046). For every one-point increase in the total sentiment score of these five donors, the subsequent donor leaves a message with ~0.7 more characters.

**Table 3:** The regression results of donation behavior.

	**Dependent variable**			
	**MessLen**	**Sentiment**	**log(DonAmt)**	**Anonymity**
	***OLS***	***OLS***	***OLS***	***Logit***
	**(1)**	**(2)**	**(3)**	**(4)**
log(DonAmt5)	0.828 (0.560)	−0.016 (0.018)	0.123^***^ (0.048)	0.048 (0.089)
MessLen5	0.004 (0.018)	0.0003 (0.001)	−0.001 (0.001)	−0.003 (0.003)
Anonymity5	0.017 (0.283)	0.010 (0.010)	−0.040 (0.026)	0.140^***^ (0.050)
Sentiment5	0.703^**^ (0.351)	0.032^**^ (0.013)	−0.022 (0.032)	0.098 (0.066)
Intercept	−1.334 (4.049)	0.408^***^ (0.133)	4.258^***^ (0.344)	−1.428^**^ (0.654)
Observations	1,481	1,481	1,481	1,481
*R*_2_	0.008	0.010	0.007	
F Statistic	2.972^**^	3.609^***^	2.736^**^	
Log Likelihood				−923.925

^***^, ^**^, and ^*^ denote statistical significance at the 0.01, 0.05, and 0.1 level. The coefficient values represent unstandardized regression coefficients. Robust standard errors are in parentheses.

Furthermore, the results in column (2) show that the sentiment scores of the last five donors not only influence the message length of the subsequent donor but also have a significant positive impact on the sentiment score of his or her message (*p*-value = 0.016). In other words, when a donor opens the fundraising platform, he or she can see the messages of the previous five donors. If the donor sees messages with more positive sentiment, the donor is more likely to leave a message with positive sentiment. Thus, as we infer in the previous section, positive sentiment is contagious, and positive sentiment's reinforcement effect is confirmed here. In other words, Hypothesis 1 is partially verified.

Column (3) shows the impact of historical donation information on the subsequent donation amount. The donation amounts of the last five people have a significant positive impact on the donation amount of a subsequent donor (*p*-value = 0.010). This result confirms Hypothesis 2: donors adjust their donation amount based on the donation amounts of others, which reflects their compliance with this descriptive social norm and is consistent with a series of studies drawing the conclusion that information about the donation amounts of previous donors increase individuals' donation amounts.

However, the messages of previous donors and choices of anonymity did not have a significant direct impact on the amount of money donated by subsequent donors, as shown in column (3). While we did not find evidence that more positive recent messages can inspire people to donate more, we believe that the messages of donors are influenced by the messages of other donors.

The results in column (4) confirm Hypothesis 3 from one perspective. These results show how the donation information of the latest five donors affects the choice of anonymity of the subsequent donor. The length of the previous donors' messages, sentiment scores, and donation amount had no significant effect on the subsequent donor's choice of anonymity, but whether the previous donors chose to remain anonymous significantly affected the subsequent donor's decision (*p*-value = 0.005). The coefficient of *Anonymity*5, β_3_, is positive; that is, when a donor observes that more previous donors chose to remain anonymous, the donor is more likely to choose to remain anonymous, and vice versa. This result suggests that people tend to imitate the actions of people before them. This is another form of conforming to social norms.

**Figure 3:**
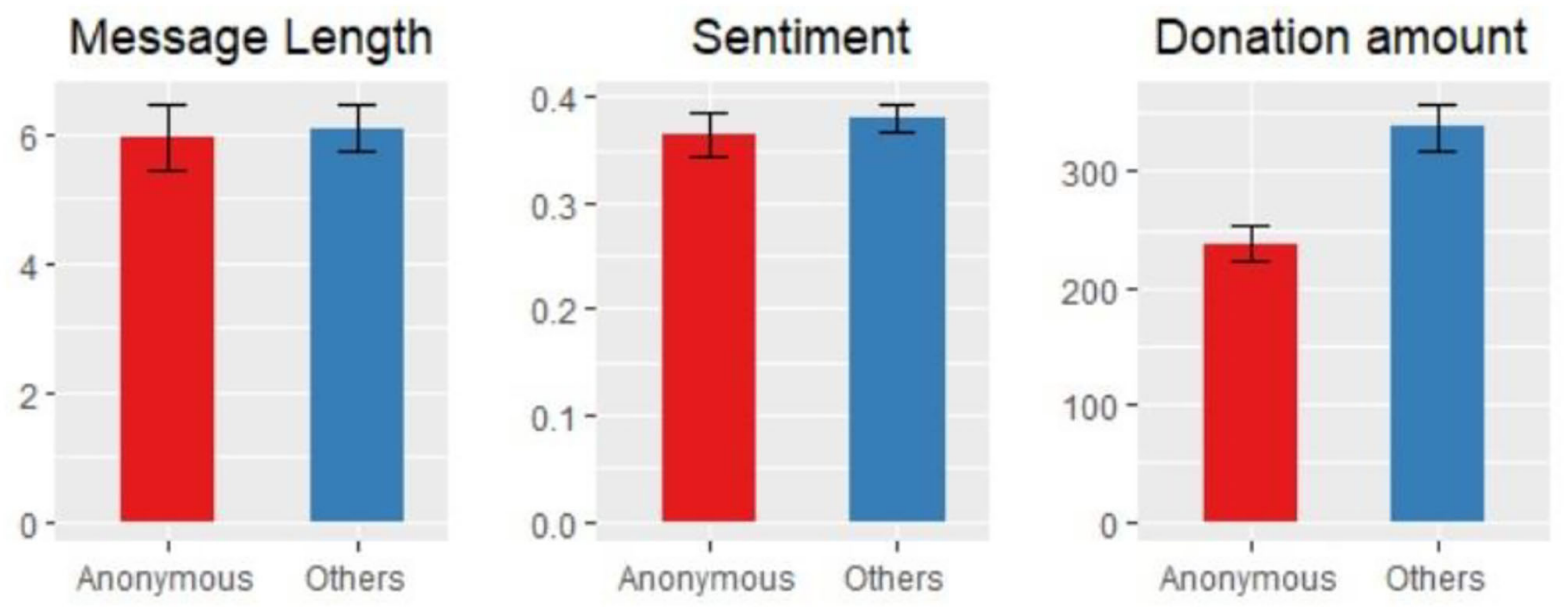
Donation behavior of anonymous and non-anonymous donors (histograms represent arithmetic means; error bars represent standard errors).

Next, we separately assessed the donation behaviors of anonymous and non-anonymous donors, and the results are shown in Figure 3.

We compared the behaviors of anonymous and non-anonymous donors. Forty-Seven and Forty-Nine percentage of anonymous and non-anonymous donors left messages, respectively. In the left panel of Figure 3, the red bar shows that the average length of messages from anonymous donors is 5.95, and the blue bar shows that the average length of messages from anonymous donors is 6.08. No significant difference was observed between the two lengths (*p*-value = 0.42; all tests reported within the text are Wilcoxon rank-sum tests). The middle panel of Figure 3 shows a similar result for the sentiments of anonymous and non-anonymous messages: no statistically significant difference is observed (*p*-value = 0.56). That is, anonymous and non-anonymous donors do not write messages with different content.

In the right panel of Figure 3, the left bar shows the average donation of anonymous donors, and the right bar shows the average donation of non-anonymous donors. A significant difference was found (*p*-value < 0.01): the average anonymous donation was 237.95 Yuan, and the average non-anonymous donation was 338.19 Yuan. This result supports Hypothesis 3: anonymous donations are smaller than non-anonymous donations. Our results regarding anonymous behaviors are consistent with those of many studies (Soetevent, 2005; Alpizar et al., 2008; Vesely and Klöckner, 2018; Firmansyah and Pratama, 2021). One possible explanation is that people attach great importance to evaluations from others and hope to be positively viewed as responsible people, especially in China, a country with a collectivistic culture. Thus, donors who do not have the ability or willingness to donate more than the socially accepted amounts in their mind will tend to remain anonymous.

### Robustness Check: Using the MR Sentiment Score

This section uses the MR sentiment score as the sentiment variable instead of the NLP sentiment score used in Table 3 to conduct regressions in Table 3. The results, shown in Table 4, are similar to those shown in Table 3.

**Table 4:** The regression results of donation behavior (MR sentiment scores).

	**Dependent variable**			
	**MessLen**	**Sentiment**	**log(DonAmt)**	**Anonymity**
	***OLS***	***OLS***	***OLS***	***Logit***
	**(1)**	**(2)**	**(3)**	**(4)**
log(DonAmt5)	0.788 (0.560)	0.035 (0.072)	0.122^**^ (0.048)	0.042 (0.089)
MessLen5	0.008 (0.019)	−0.002 (0.002)	−0.001 (0.001)	−0.003 (0.003)
Anonymity5	0.019 (0.284)	0.041 (0.038)	−0.040 (0.026)	0.139^***^ (0.050)
Sentiment5	0.131 (0.099)	0.052^***^ (0.014)	0.001 (0.009)	0.024 (0.018)
Intercept	−1.466 (4.008)	1.576^***^ (0.532)	4.282^***^ (0.346)	−1.501^**^ (0.664)
Observations	1,481	1,481	1,481	1,481
*R*_2_	0.004	0.011	0.004	
F Statistic	2.455^**^	5.196^***^	2.640^**^	
Log Likelihood				−924.075

^***^, ^**^, and ^*^ denote statistical significance at the 0.01, 0.05, and 0.1 level. The coefficient values represent unstandardized regression coefficients. Robust standard errors are in parentheses.

The results of Column (1) in Table 3 show that the sentiment scores of the last five donors have a significant positive impact on the length of the donor's message. In contrast, this positive effect disappears in Table 4 when using the MR sentiment score to replace the NLP sentiment score. All the results of Columns (2), (3), and (4) in Table 4 confirm the robustness of those in Table 3.

### Robustness Check: Time Trend Controlled

This section shows regressions that control for the time trend, and similar results are shown in Table 5.

**Table 5:** The regression results of donation behavior (time trend controlled).

	**Dependent variable**			
	**MessLen**	**Sentiment**	**log(DonAmt)**	**Anonymity**
	***OLS***	***OLS***	***OLS***	***Logit***
	**(1)**	**(2)**	**(3)**	**(4)**
log(DonAmt5)	1.098^*^ (0.581)	−0.011 (0.019)	0.110^**^ (0.048)	0.078 (0.090)
MessLen5	−0.003 (0.018)	0.0001 (0.001)	−0.001 (0.001)	−0.003 (0.003)
NoMess5	1.567 (1.624)	0.019 (0.052)	0.169 (0.155)	0.328 (0.290)
Anonymity5	−0.050 (0.283)	0.009 (0.010)	−0.036 (0.026)	0.133^***^ (0.049)
Sentiment5	0.832^**^ (0.355)	0.034^**^ (0.014)	0.035 (0.034)	0.125* (0.068)
Intercept	−3.436 (4.523)	0.359^**^ (0.143)	4.579^***^ (0.367)	−1.545^**^ (0.712)
Control for the time trend	Yes	Yes	Yes	Yes
Observations	1,481	1,481	1,481	1,481
*R*_2_	0.016	0.012	0.011	
F Statistic	3.943^***^	2.900^***^	2.823^***^	
Log Likelihood				−921.566

^***^, ^**^, and ^*^ denote statistical significance at the 0.01, 0.05, and 0.1 level. The coefficient values represent unstandardized regression coefficients. Robust standard errors are in parentheses.

Except for the NoMess5 dummy variable and controlling for the time trend, the dependent and independent variables of Table 5 are the same as those in Table 3. Table 5 shows results that are similar to those shown in Table 3. Furthermore, NoMess5 means that there is no message left by the previous five donors, and it has no significant impact on the dependent variables.

When historical messages contain more positive sentiment, subsequent donors are more likely to be affected by the positive sentiment and to leave longer and more positive texts, thus forming a virtuous cycle with a trend of spreading positive sentiment.

**Figure 4:**
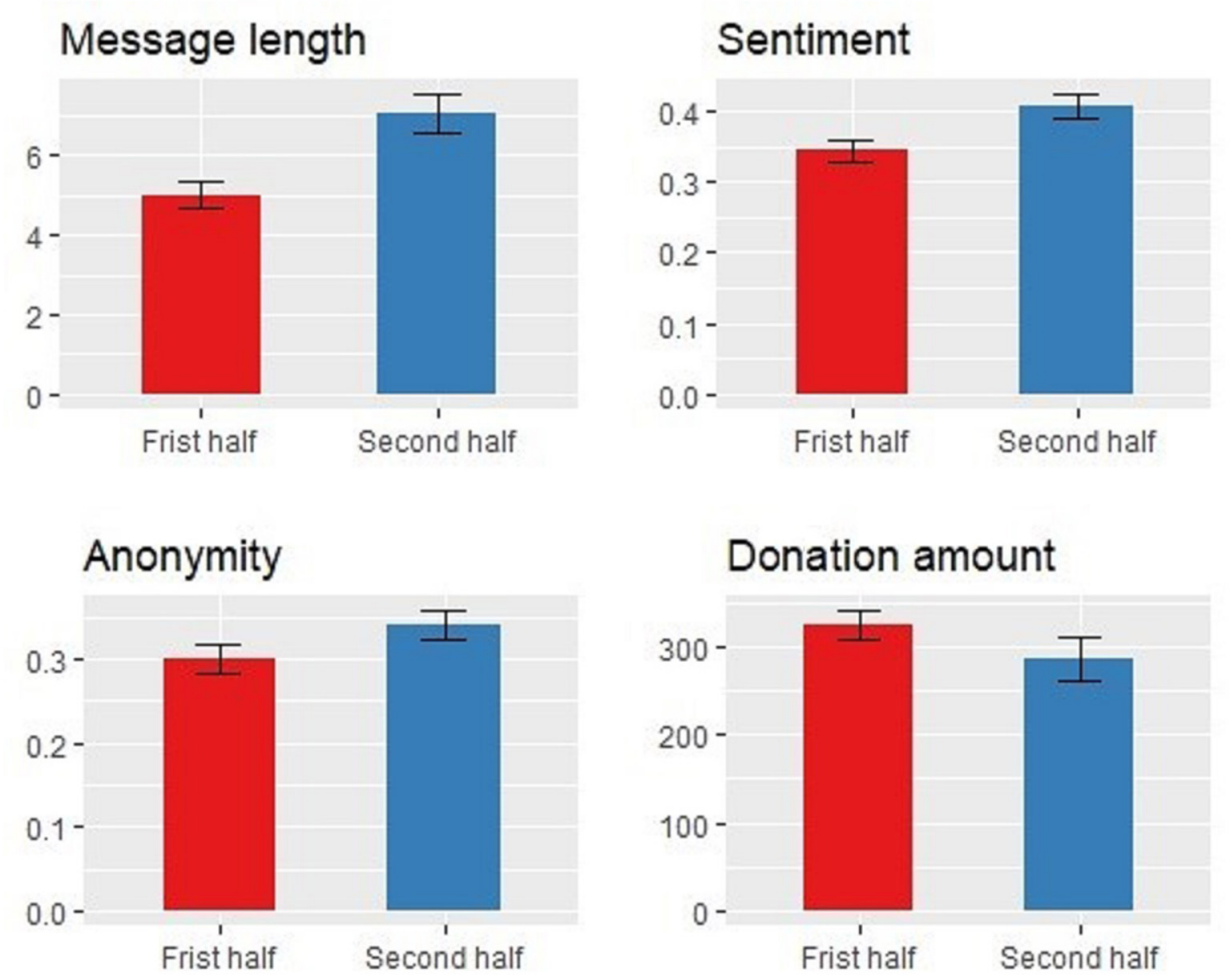
Trends in the donation sequence (histograms represent arithmetic means; error bars represent standard errors).

Figure 4 shows comparisons between the first and second halves of the donation sequence. The message length of the first half is 5.02, which is significantly less than that of the second half, 7.05 (*p*-value < 0.01). The sentiment score had the same significant trend, from 0.34 in the first half to 0.41 in the second half (*p*-value < 0.01). However, anonymity did not have a significant trend (*p*-value = 0.11), and the number of donations had an opposite significant downward trend from the first half (325.00) to the second half (287.11) (*p*-value < 0.01).

### Placebo Test: Using the Information of the 100 Previous Donors

This section shows the results of the placebo test. Instead of the main regression using the information of the past five periods, the regression uses the information of the placebo test as the independent variable and finds no results that are significant at the 5% level.

In Table 6, the independent variable *log*(*DonAmt*__*m*_100_) is the logarithm of the average donation of the last 100 donors before the current donor. *MessLen*__*m*_100_ is the average message length of these 100 donors. We average the dummy variable values of whether the latest 100 donors are anonymous to obtain the variable *Anonymity*__*m*_100_. In the same way, the average sentiment scores of the 100 people who left messages before the current donor are calculated as *Sentiment*__*m*_100_. The dependent variables are the same as in Table 3. The VIFs (variance inflation factors) of *log*(*DonAmt*__*m*_100_), *MessLen*__*m*_100_, *Anonymity*__*m*_100_ and *Sentiment*__*m*_100_ are 1.362, 3.050, 1.580, and 3.967, respectively, which represent a low level of multicollinearity.

**Table 6:** The placebo regression results of donation behavior.

	**Dependent variable**			
	**MessLen**	**Sentiment**	**log(DonAmt)**	**Anonymity**
	***OLS***	***OLS***	***OLS***	***Logit***
	**(1)**	**(2)**	**(3)**	**(4)**
*log*(*DonAmt*_*m*100_)	0.252 (1.790)	−0.009 (0.067)	0.192 (0.168)	0.271 (0.352)
*MessLen*_*m*100_	0.266 (0.463)	0.009 (0.017)	0.036 (0.040)	0.012 (0.081)
*Anonymity*_*m*100_	1.940 (6.158)	0.136 (0.261)	0.695 (0.665)	0.557 (1.333)
*Sentiment*_*m*100_	11.795 (10.966)	0.339 (0.388)	1.753^*^ (0.959)	3.733^*^ (1.974)
Intercept	4.204 (11.599)	0.294 (0.437)	7.260^***^ (1.076)	3.442 (2.283)
Observations	1,381	1,381	1,381	1,381
*R*_2_	0.003	0.005	0.020	
F Statistic	0.925	1.630	7.135^***^	
Log Likelihood				–862.418

^***^, ^**^, and ^*^ denote statistical significance at the 0.01, 0.05, and 0.1 level. The coefficient values represent unstandardized regression coefficients. Robust standard errors are in parentheses.

## Discussion and Conclusion

Our findings extend the results of previous studies. The social norm effect reveals that donors tend to mimic other people's donations after observing how much they donate. This paper conducts a broader study on compliance with social norms and finds that donors' imitation of others is not limited to the amount of money donated but also includes their choice of anonymity and the positive sentiment expressed in their messages. This research has the following highlights:

First, the online donation scenario considered in this paper has much stronger environmental control than on-site donations. In an on-site donation, the information received by each donor may vary greatly. In this online fundraising platform, donors donated through mobile phones, and all donors saw the same page, the same introduction and the same donation environment. In other words, the information structure observed by each donor was consistent. Additionally, the donors had similar donation reasons and similar educational backgrounds because the donation campaign was initiated by the alumni association and donations were given to alumni. Last, the anonymity of online donations is more secure than that of offline donations.

Second, this paper uses natural language processing and manual scoring to evaluate the positive sentiment degree of donors' messages and finds that positive sentiment in messages is infectious, leading to the spread of positive sentiment. Chen et al. (2021) suggest that emotional elements are also worth considering in a charitable setting, and previous studies have ignored exploring the connotative emotional cues inside the texts or pictures presented by online charity projects. Based on this idea, we try to examine the effects of previous donors' messages on subsequent donors' behaviors. Although our results provide no evidence that the sentiment of the message significantly impacts donation amounts, this paper provides several references for researchers to explore the effects of the message on donation behaviors, including donation participation rates, donation amounts, and other behaviors.

Third, the findings in this paper provide ideas for the design of a fundraising platform. To improve the effectiveness of fundraising projects, we suggest that historical donation amounts be disclosed. In particular, several pieces of information with the highest donation amount can be displayed on the top of the donation page to motivate subsequent donors. The choice to remain anonymous could be an option, but platform developers should consider whether to show anonymous donations to others.

One concern regarding the conclusion of this paper is the particularity of donations from the WHU Alumni Association. However, online donations generally occur in groups with specific relationships, and we will conduct further research on other types of group donations in the future. As another concern, this paper assumes that the appearance of online donors is completely random. This assumption cannot be verified in the empirical data, which may cause problems of endogeneity. Additionally, this paper does not indicate whether the positive sentiment in messages can attract more potential donors. If it can, we then can explain why COVID-19-related donations are shared more on social media, have a higher total amount, and last longer than others. In the future, lab and field experiments with structures similar to crowdfunding donation can be used for further research.

## Data Availability Statement

The raw data supporting the conclusions of this article will be made available by the authors, without undue reservation.

## Author Contributions

YL collected and cleaned all the data, and undertook the sentimental analysis. YP conducted the analysis and wrote the paper. LW contributed the ideas and was in charge of the submission and the revision of the paper. All authors contributed to the article and approved the submitted version.

## Funding

The authors gratefully acknowledge the National Natural Science Foundation of China (Grant No. 72173093).

## Conflict of Interest

The authors declare that the research was conducted in the absence of any commercial or financial relationships that could be construed as a potential conflict of interest.

## Publisher's Note

All claims expressed in this article are solely those of the authors and do not necessarily represent those of their affiliated organizations, or those of the publisher, the editors and the reviewers. Any product that may be evaluated in this article, or claim that may be made by its manufacturer, is not guaranteed or endorsed by the publisher.

## Footnotes

^1^Charitable Giving Report 2021 from www.blackbaud.com.

^2^Wuhan University is the largest and most influential university in Wuhan. Overseas alumni of Wuhan University donated a large number of medical supplies in the early stage of the COVID-19 epidemic. At the end of March, the epidemic had been effectively controlled in Wuhan, but the epidemic in North America was widespread. The Chinese alumni launched this online event to raise funds to purchase medical supplies (e.g., medical masks) for North American alumni.

^3^Sentiment analysis is the use of natural language processing, text analysis, computational linguistics, and biometrics to systematically identify, extract, quantify, and study affective states and subjective information. Sentiment analysis is widely applied to the voice of the customer materials such as reviews and survey responses, online and social media, and healthcare materials for applications that range from marketing to customer service to clinical medicine (https://en.wikipedia.org/wiki/Sentiment_analysis).

^4^On the donation platform, donors' self-reported names are displayed to the public if they do not choose to be anonymous. Based on the data, we find all the non-anonymous donors showed their real names instead of nicknames.

^5^Because the page contains a brief introduction to the donation, the length of the actual donation page is so long that one cannot see the anonymous option when one first chooses the donation amount. This is one of the reasons why we believe that the donation amount will affect anonymity.

^6^The value output of snowNLP ranges from 0 to 1, with 0 representing negative sentiment and 1 representing positive sentiment. Based on previous studies, snowNLP is often used to analyze messages or texts that express individuals' subjective experience of something or their feelings, such as tourists' experience of attractions (Zhang et al., 2022) and potential borrowers' feelings toward a peer-to-peer (P2P) lending platform (Huang et al., 2021). Therefore, it is feasible to adopt snowNLP to conduct text analysis. Furthermore, to verify the significance of the snowNLP sentiment scores, we recruited subjects to manually score the messages of donors (details are displayed as follows).

^7^We do not directly use the word “positive” in the questionnaire because we believe that all messages in this donation are not negative. The translation of the questionnaire is in the Appendix.

^8^Unless otherwise specified, the sentiment scores mentioned in this paper refer to the NLP sentiment scores rated by snowNLP.

## Appendix: The Introduction to Manual Rating

**Questionnaire task:** Please rate the donation message of a previous donation project.

**[Donation Project Introduction]** In 2020, with the spread of COVID-19, the Wuhan University Beijing Alumni Association, under the call of the Wuhan University Alumni Association, responded to North American alumni's appeal for material help and raised funds for overseas alumni to purchase masks, protective suits and other protective resources.

A vast number of alumni and caring people enthusiastically supported this project and lent a helping hand (Note: The donation project was launched on March 25, 2020, when the domestic pandemic was basically under control and the overseas pandemic began to break out).

This questionnaire contains 116 questions; each question stem is a message left by a previous donor when donating. Please rate the emotional strength of each message, with 1 point being the weakest and 7 points being the strongest (To protect the donors' privacy, we replace the personal information in the message with [XXX]).
